# Diagnosis of Chronic Pulmonary Aspergillosis

**DOI:** 10.4269/ajtmh.23-0053b

**Published:** 2023-04-17

**Authors:** Felix Bongomin, Conrad K. Muzoora, Davidson H. Hamer

**Affiliations:** Department of Medical Microbiology and Immunology Faculty of Medicine Gulu University Gulu, Uganda E-mail: drbongomin@gmail.com; Department of Internal Medicine Faculty of Medicine Mbarara University of Science and Technology Mbarara, Uganda E-mail: conradmuzoora@yahoo.com; Department of Global Health School of Public Health Boston University Boston, Massachusetts E-mail: dhamer@bu.edu

Dear Editor,

We read with interest the study by Oliveira and colleagues recently published in the *Journal*.^1^ The authors retrospectively investigated the diagnostic performance of various laboratory tests for different subtypes of chronic pulmonary aspergillosis (CPA). The study results showed that bronchoalveolar lavage galactomannan, serology by immunodiffusion test, and histology had the highest “sensitivity” for diagnosing CPA. The counterimmunoelectrophoresis (CIE) titers differed significantly between the different subtypes of CPA, with higher titers seen in the chronic fibrosing pulmonary aspergillosis and subacute invasive aspergillosis (SAIA) subtypes. Furthermore, C-reactive protein values were generally low, but higher in the SAIA subtype.

These study results provide insight into the diagnostic performance of different tests in diagnosing CPA subtypes and can be useful for clinicians in determining the appropriate tests to use for each subtype of CPA. The authors have already highlighted several limitations of their study such as small sample size, very few cases with histological confirmation of diagnosis, lack of clinical information, single-center, retrospective design, and lack of mycological identification of *Aspergillus* species. All this information would have helped to better understand the diagnostic performance of the tests.

We would like to highlight further limitations to this study. First, there was no control group. The study did not include a control group without CPA, which would have provided a benchmark for comparison and improved the interpretability of the results. Use of diseased and/or healthy controls are key for diagnostic test evaluations and have been previously reported in several studies demonstrating diagnostic performance of tests for CPA.^2^^,^^3^ Second, in line with the above, the use of the term “sensitivity” may be inappropriate, as this implies the ability of the test to detect cases among both diseased and non-diseased populations. Therefore, it is likely that the authors are reporting on positivity rates of the various tests among CPA cases. A complete report of diagnostic accuracy, sensitivity, specificity, and positive and negative predictive values is impossible without including a control group. Third, the study used a single cut-off value for some tests, such as C-reactive protein and bronchalveolar galactomannan, which might affect the accuracy of the results. Ideally, this study would have been executed using a receiver operating characteristics curve analysis evaluating different cut-off values with their corresponding areas under the curve. Again, this is impossible without a control group.

There is no single gold-standard test for the diagnosis of CPA.^4^ The diagnosis of CPA is based on a combination of clinical, radiological, immunological, and mycological characteristics as outlined in the European Society for Clinical Microbiology and Infectious Diseases and the European Respiratory Society guidelines,^5^ with modifications for resource-limited settings.^6^ Histology is not considered a gold-standard for the diagnosis of CPA, as mentioned by the authors. It is also inappropriate to evaluate the diagnostic performance of a test which by itself was included in the diagnostic criteria of the cases.

In conclusion, the study provides valuable information on the diagnostic performance of various tests for CPA. However, the results should be interpreted with caution given the major limitations of the study methods and results. Further research is needed to validate these findings and to provide more robust and generalizable results.
